# Removal of the Basic and Diazo Dyes from Aqueous Solution by the Frustules of *Halamphora* cf. *salinicola* (Bacillariophyta)

**DOI:** 10.3390/md21050312

**Published:** 2023-05-19

**Authors:** Aleksandra Golubeva, Piya Roychoudhury, Przemysław Dąbek, Oleksandra Pryshchepa, Paweł Pomastowski, Jagoda Pałczyńska, Piotr Piszczek, Michał Gloc, Renata Dobrucka, Agnieszka Feliczak-Guzik, Izabela Nowak, Bogusław Buszewski, Andrzej Witkowski

**Affiliations:** 1 Institute of Marine and Environmental Sciences, University of Szczecin, Mickiewicza 16a, 70-383 Szczecin, Polandprzemyslaw.dabek@usz.edu.pl (P.D.); 2 Centre for Modern Interdisciplinary Technologies, Nicolaus Copernicus University, Wileńska 4, 87-100 Toruń, Poland; 503144@doktorant.umk.pl (O.P.); p.pomastowski@umk.pl (P.P.); 3 Department of Inorganic and Coordination Chemistry, Faculty of Chemistry, Nicolaus Copernicus University, Gagarina 7, 87-100 Toruń, Poland; 296600@stud.umk.pl (J.P.); piszczek@umk.pl (P.P.); 4 Faculty of Materials Science and Engineering, Warsaw University of Technology, Wołoska 141, 02-507 Warsaw, Poland; michalgloc@wp.pl (M.G.); renata.dobrucka@pw.edu.pl (R.D.); 5 Department of Industrial Products and Packaging Quality, Institute of Quality Science, Poznań University of Economics and Business, al. Niepodległości 10, 61-875 Poznan, Poland; 6 Department of Applied Chemistry, Faculty of Chemistry, Adam Mickiewicz University, Uniwersytetu Poznańskiego 8, 61-614 Poznań, Poland; agnieszka.feliczak-guzik@amu.edu.pl (A.F.-G.); nowakiza@amu.edu.pl (I.N.); 7 Department of Environmental Chemistry and Bioanalysis, Faculty of Chemistry, Nicolaus Copernicus University, Gagarina 7, 87-100 Toruń, Poland; bbusz@umk.pl; 8 Prof. Jan Czochralski Kuyavian-Pomeranian Research & Development Centre, Krasińskiego 4, 87-100 Toruń, Poland

**Keywords:** biosilica, adsorption, congo red, crystal violet, malachite green

## Abstract

Industrial wastes with hazardous dyes serve as a major source of water pollution, which is considered to have an enormous impact on public health. In this study, an eco-friendly adsorbent, the porous siliceous frustules extracted from the diatom species *Halamphora* cf. *salinicola*, grown under laboratory conditions, has been identified. The porous architecture and negative surface charge under a pH of 7, provided by the various functional groups via Si–O, N–H, and O–H on these surfaces, revealed by SEM, the N_2_ adsorption/desorption isotherm, Zeta-potential measurement, and ATR-FTIR, respectively, made the frustules an efficient mean of removal of the diazo and basic dyes from the aqueous solutions, 74.9%, 94.02%, and 99.81% against Congo Red (CR), Crystal Violet (CV), and Malachite Green (MG), respectively. The maximum adsorption capacities were calculated from isotherms, as follows: 13.04 mg g^−1^, 41.97 mg g^−1^, and 33.19 mg g^−1^ against CR, CV, and MG, respectively. Kinetic and isotherm models showed a higher correlation to Pore diffusion and Sips models for CR, and Pseudo-Second Order and Freundlich models for CV and MG. Therefore, the cleaned frustules of the thermal spring-originated diatom strain *Halamphora* cf. *salinicola* could be used as a novel adsorbent of a biological origin against anionic and basic dyes.

## 1. Introduction

Diatoms (*Bacillariophyceae*) are photosynthetic unicellular eukaryotes with unique three-dimensional perforated shells surrounding the cells—the so-called frustules. They are responsible for 20% of global primary production and play an essential role in the silicon cycle, due to their ability to uptake silicic acid from the environment and deposit it within their cell walls in the form of opal (SiO_2_ nH_2_O) [1]. Their adaptable metabolism allows them to grow in any kind of environment, from marine and fresh waters to thermal geysers and polar glaciers [2]. In recent years a great selection of various diatom taxa have been studied for their industrial potential [3].

*Halamphora* spp. has gained attention due to its ability to accumulate a high amount of triglycerides (TAG) for biodiesel production [4,5,6,7], fatty acids for the aquaculture [8,9] and cosmetics industries [10], as well as fucoxanthin and chrysolaminarin with antioxidant, anticancer, and immunomodulatory capacities for pharmaceutical purposes [11,12]. Thus, *Halamphora* strains can be cultivated on an industrial scale for biodiesel, while wastes from lipid extraction may be purified and clean biosilica can be used in different applications. A single report of *Halamphora* spp. mediated biosynthesis of hybrid SiO_2_–Ag nanodendrites is available [13], while other pennate diatoms have shown promising results in metal nanoparticles biosynthesis (*Gedaniella flavovirens*, *G*. *mutabilis*, and *Nanofrustulum shiloi*) [14,15], drug delivery (*Amphora subtropica* and *Nitzschia* sp.) [16,17], biosensing (*Amphora* sp., *Pinnularia* sp., and *Pseudostaurosira trainorii*) [18,19,20,21], and heavy metal remediation (*Nitzschia* spp., *Navicula* spp., and *Pheodactylum tricornutum*) [22,23]. Furthermore, the growth rate and morphology of *H. veneta* have been proposed for the toxicological assessment of copper and mercury in aquatic environments [24]. 

Water pollution is recognized to have an enormous impact on public health, and the discharge from dye-based industries serves as a major source of contamination: up to 15% of the total dyes used in the textile industry remain untreated and are lost in emissions [25]. Synthetic dyes, released into waters by textile, leather, paper, printing, cosmetics, plastic, and pharmaceutical industries, usually resist ordinary treatment techniques and can have a negative influence on both aquatic organisms and human health [26]. Dyes are classified according to their chemical structure and application: acidic, basic, direct, reactive, etc. [27]. Crystal Violet (CV) and Malachite Green (MG) are water soluble basic cationic dyes and belong to the triphenylmethane type, whereas Congo Red (CR) is an acidic anionic diazo dye, based on a benzidine—a well-known human carcinogen [28]. These dyes are used as models for evaluation of the adsorption ability of different adsorbents. A variety of techniques have been used for the removal of CR, CV, and MG from aqueous solutions: photocatalytic degradation [29,30,31], sonication [32,33,34], ozonation [35,36,37], oxidation [38,39,40], Fenton process [41,42,43], and biological degradation [44,45,46]. However, all these approaches have limitations in terms of efficiency, cost, and design [47]. The most effective and widely used treatment for dye removal from aqueous solutions is adsorption. Various adsorbents of diverse origins have been used in the basic and anionic dye adsorption processes, namely activated carbon [48,49,50], nanoparticles [51,52,53], nanotubes [54,55,56], waste materials [57,58,59], naturally occurring materials such as clays [60,61,62], zeolites and diatomite [63,64,65], and bio-adsorbents [66,67,68]. To the best of our knowledge, reports about the utilization of pure diatom frustules in the dye adsorption procedure are limited [69,70]. The main difference between the purified frustules from diatom culture and diatomaceous earth is the presence of organic compounds on the frustule surface, whose functional groups play an important role in the adsorption process. 

This study presents a novel eco-friendly adsorbent of biological origin, derived from the diatom strain *Halamphora* cf. *salinicola* Levkov and Díaz, produced under laboratory conditions, characterized morphologically and functionally for the first time, and which could perform against anionic and basic dyes with a high removal efficiency and adsorption capacity.

## 2. Results

### 2.1. Batch Cultivation of SZCZM1454 H. cf. salinicola

**Figure 1:**
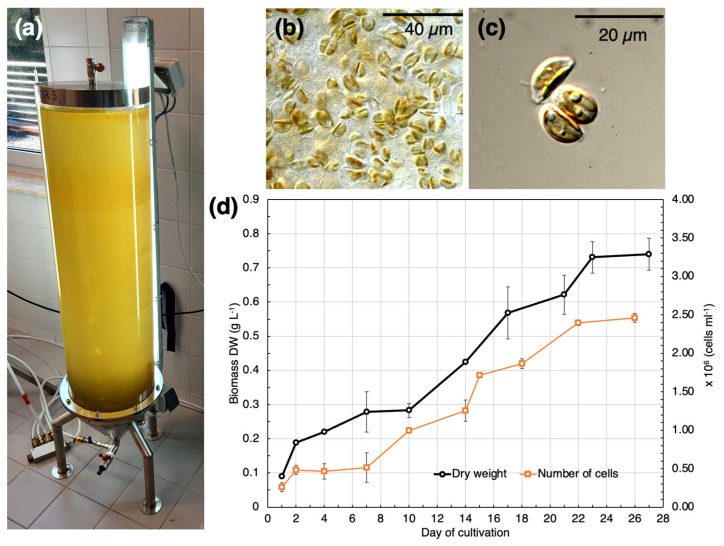
Growth of SZCZM1454 *H*. cf. *salinicola*: (**a**) photobioreactor (PBR) for batch cultivation; (**b**,**c**) cell culture under light microscope (LM) with different magnification; and (**d**) growth curve for the dry biomass yield and the number of cells.

Figure 1 presents the growth of strain SZCZM1454 *H*. cf. *salinicola*. From the second day of cultivation this strain grew exponentially until it reached its maximum on the 22nd day. The maximum biomass yield DW (m_max_ = 0.73 ± 0.047 g L^−1^) and the maximum cell density (C_max_ = 2.46 ± 0.004 × 10^6^ cells per ml) were observed after 23 days. By the end of the cultivation period, uptake of nitrate was 79.5% and silicate was 30% (0.7 mM and 0.033 mM of nitrate and silicate, respectively), whereas phosphate uptake was the highest at 99.9% (0.0359 mM). The maximum specific growth rate was calculated as 0.081 d^1^ (R^2^ = 0.935) for the biomass yield and 0.113 div d^−1^ (R^2^ = 0.949) for cell density. After the 22 days, the biomass yield and cell density had not changed for several days, which indicated the start of the stationary growth phase. 

Table 1 shows the dry biomass yield of strain SZCZM1454 *H*. cf. *salinicola* grown under a variety of nutrient concentrations, salinity, illumination intensity, and temperature. The increased concentration of silicates in the f/2 medium resulted in a significant change in the dry biomass yield (one-way ANOVA, *p* = 0.0002): the highest biomass accumulation was observed for samples grown in medium with 20 times higher silicate concentration (Turkey HSD, *p* = 0.001 between 2.12 and 0.11, 0.53, or 1.06 mM; and *p* = 0.035 between 2.12 and 1.59 mM). Likewise, a significant difference in the biomass yield was observed for samples cultivated in a medium with a higher salinity (one-way ANOVA, *p* = 0.043; Turkey HSD, *p* = 0.0082 between 20 ppt and 45 ppt). Furthermore, a higher cultivation temperature (30 °C) of cultivation resulted in a higher biomass yield (one-way ANOVA, *p* = 0.006; Turkey HSD, *p* = 0.0063, 0.0113 between 30 °C and 15 °C, 20 °C, respectively), whereas low light intensity (10 µmol s^−1^ m^−2^) decreased the biomass accumulation in comparison with a higher illumination (one-way ANOVA, *p* = 0.0082; Turkey HSD, *p* = 0.0075, 0.016 between 10 and 50, 150 µmol s^−1^ m^−2^, respectively). No significant differences were observed in the biomass yield for samples grown in a medium with higher concentrations of nitrate (one-way ANOVA *p* = 0.344) and phosphate (one-way ANOVA *p* = 0.224). 

### 2.2. Characterization of the Frustule Surface 

**Figure 2:**
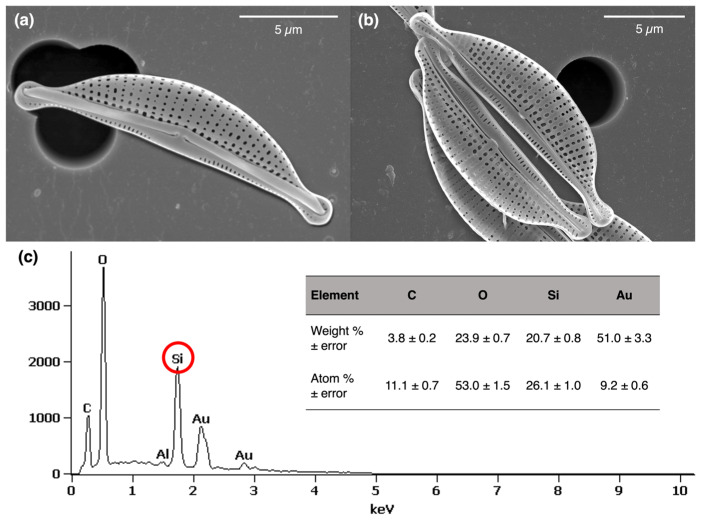
Morphology and the elemental analysis of the SZCZM1454 *H*. cf. *salinicola* frustules: (**a**,**b**) SEM images revealed the surface topography of the frustules and (**c**) the EDS spectra detected the presence of silicon (Si) on the surface of the frustules.

The SEM images (Figure 2a,b) revealed the surface topography of the SZCZM1454 *Halamphora* cf. *salinicola* frustules. The siliceous frustule is composed of two valves which are semi-lunate in shape and dorsiventral with slightly capitate apices. The dorsal margin of the valve is gently arched, and its ventral margin is straight. The length and width of the valves were measured as 14.0–19.0 mm and 3.0–4.15 mm, respectively. Pores, called areolae, are spherical in the center of the valve face and become elliptical on each side of a slit, called the raphe. The size of the areolae decrease towards the dorsal margin, and the length and width of the areolae were calculated as 0.07–0.23 mm and 0.1–0.34 mm, respectively. The areolae are arranged in rows, the transapical striae, and consist of 1–2 to 7–9 areolae. The number of striae was calculated as 39–48.

The elemental composition analysis of the frustules (Figure 2c), demonstrated by the Energy dispersive X-ray spectroscopy, demonstrated the presence of silica (Si), carbon (C), oxygen (O), and gold (Au) on the sample’s surface. The weight percentage was calculated as 20.7, 3.8, 23.9, and 51.0%, respectively, and the atomic percentage was estimated as 26.1, 11.1, 53.0, and 9.2%, respectively. Au peaks indicate a gold layer from sample preparation for SEM and EDS analyses. 

**Figure 3:**
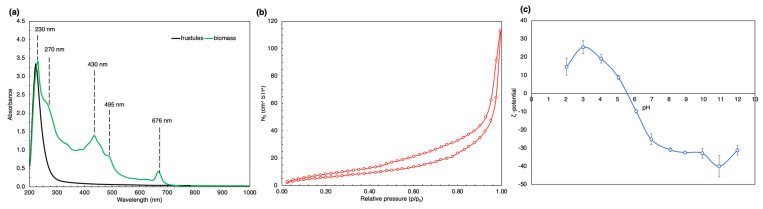
Surface characterization of the SZCZM1454 *H*. cf. *salinicola* frustules: (**a**) the UV–vis spectroscopy revealed the presence of one peak for the cleaned frustules and several peaks for the biomass; (**b**) the low temperature N_2_ adsorption/desorption isotherm; and (**c**) the zeta potential values recorded for the different pH.

Demonstrated in Figure 3a, the UV–vis spectra of the sonicated biomass (green line) showed several distinct peaks at 230, 270, 430, 495, and 676 nm. The UV–vis spectra of the sonicated frustules (black line) revealed only one distinct peak at 230 nm. 

The results of low-temperature N_2_ adsorption/desorption were analysed using the BET (Brunauer–Emmett–Teller) method to determine the specific surface area of the frustules and the BJH (Barrett–Joyner–Halenda) algorithm was used for the calculation of pore volume and diameter, presented in Table 2. Figure 3b shows the N_2_ adsorption/desorption isotherm. 

Figure 3c presents the measured zeta potential values as a function of pH. The biosiliceous frustules revealed the positive charge in a pH range of 2–5.5, and at a pH higher than 7, the negative charge.

**Figure 4:**
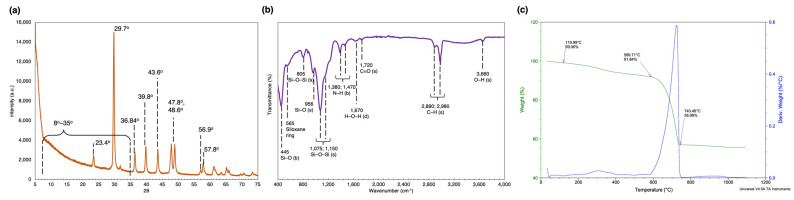
Characterization of the SZCZM1454 *H*. cf. *salinicola* frustules: (**a**) the X-ray powder diffractogram; (**b**) the FTIR spectra; and (**c**) the TGA/DTA analysis.

On the X-ray powder diffractogram (Figure 4a) the signals at 2θ ≈ 23.4°, 29.7°, 36.84°, 39.8°, 43.6°, 47.8°, 48.6°, 56.9°, and 57.8° can be distinguished. Furthermore, there is a continuous elevation in the 2θ range from 10° to 35°. 

The ATR-FTIR spectra, presented in Figure 4b, demonstrates several distinct peaks, confirming the existence of O–H, C–H, C = O, N–H, and Si–O functional groups on the surface of the frustules: 3660 and 1670 cm^−1^; 2980 and 2890 cm^−1^; 1720 cm^−1^; 1470 and 1380 cm^−1^; and 1150, 1075, 956, 805, and 445 cm^−1^, respectively.

Thermogravimetric analysis (Figure 4c) showed the overall weight loss of the sample as almost 45%. The TGA curve revealed a gradual decrease in the temperature range of 0–600 °C with an overall mass loss of nearly 18.16%. Using differential thermal analysis (DTA) it was possible to distinguish two main steps in the relevant range. The first one that ended at nearly 120 °C was about 1%. The second, in the range of 150–400 °C, showed a mass loss of 7.2%. At temperatures higher than 600 °C, drastic weight loss occurred. 

### 2.3. Batch Adsorption Study 

**Figure 5:**
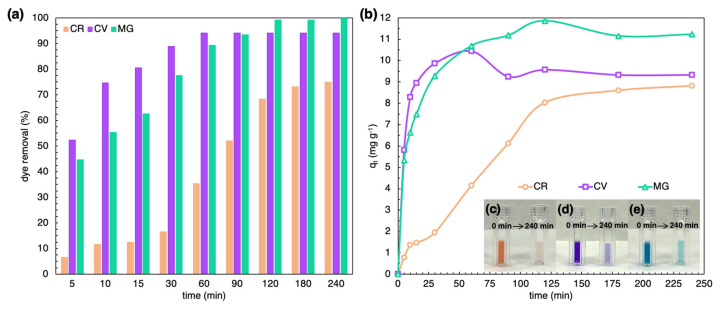
Discoloration of CR, CV, and MG solution after exposure to the SZCZM1454 *H*. cf. *salinicola* frustules: (**a**) dye removal efficiency and (**b**) adsorption capacity. Changes of solutions’ color after 240 min for (**c**) CR, (**d**) CV, and (**e**) MG (initial concentration of dye–20 mg L^−1^, adsorbent dosage–20 mg, pH–7, temperature–20 °C, time–5–240 min).

The frustules of SZCZM1454 *H*. cf. *salinicola* showed good removal activity for the diazo dyes—Congo Red (CR), and the basic dyes—Crystal Violet (CV) and Malachite Green (MG). Figure 5c–e shows visible discoloration of the aqueous dye solutions after 240 min of exposure. The UV–vis measurements confirmed the elimination of dyes over time—Figure 5a shows rapid removal of CV in the first five minutes of exposure, followed by a gradual increase until the system reached equilibrium at 60 min. The removal of MG shows a similar pattern, but at a slower rate with 50% removal achieved after 10 min of exposure, and the system only reached equilibrium after 120 min. CR removal gradually increased after 30 min until it reached equilibrium after 180 min. The frustules of SZCZM1454 *H*. cf. *salinicola* exhibited the highest removal efficiency against the basic dyes with 94.02% and 99.81% removal of CV and MG, respectively, after 60 min and 240 min. However, their efficiency against the diazo dye was lower, with 74.9% removal of CR after 240 min of exposure. The change in adsorption capacity over time is presented in Figure 5b. The adsorption capacity is estimated to be 8.81, 10.44, and 11.86 mg g ^−1^ for CR, CV, and MG, respectively.

Linear kinetic and diffusion models (Figure S1) were applied to investigate possible mechanisms behind sorption of different types of dyes onto SZCZM1454 *H*. cf. *salinicola*. Table 3 shows the kinetic parameters for each model. The closest to a unity correlation coefficient R^2^ (higher than 0.93) was observed for the Pseudo-First Order, Intra particle diffusion, and Pore diffusion models for the adsorption of the diazo dye CR (Figure S1a,c,d). However, the Chi-square values were considerably lower for the Intra particle and Pore diffusion models. Figure S1b presents the Pseudo-Second Order model as the best fit for the experimental data of CV and MG adsorption onto the SZCZM1454 *H*. cf. *salinicola* frustules, with the highest correlation coefficient (higher than 0.95 and 0.99, respectively) and the lowest Chi-square value (around 3 and 0.2, respectively). 

**Figure 6:**
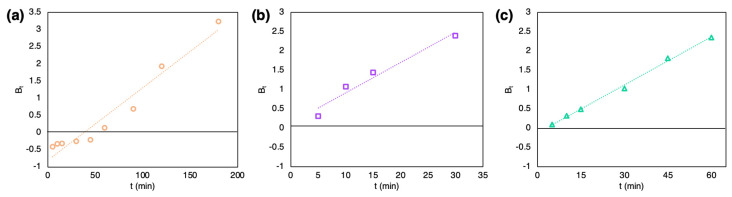
Boyd’s plot for adsorption of (**a**) CR, (**b**) CV, and (**c**) MG onto the SZCZM1453 *H*. cf. *salinicola* frustules.

To determine the main resistance to mass transfer, the Boyd’s model was used (Figure 6). Graphs for all three dyes were straight lines (correlation coefficients higher than 0.95), although only the plot for CR adsorption passed through the origin (Figure 6a), while CV and MG (Figure 6b,c) did not.

**Figure 7:**
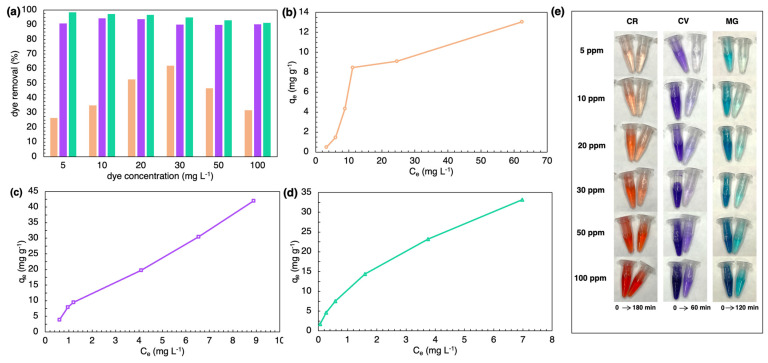
Influence of initial dye concentration on removal: (**a**) dye removal efficiency; adsorption isotherms of (**b**) CR, (**c**) CV, and (**d**) MG; and (**e**) discoloration of solutions with different dye concentrations (initial concentration of dye–5–100 mg L^−1^; adsorbent dosage–20 mg, pH–7, temperature–20 °C, time–180 min, 60 min, and 120 min for CR, CV, and MG, respectively).

Figure 7a shows the effect of different initial concentrations on removal efficiency. For the diazo dye CR, increasing the concentration of the solution resulted in a gradual increase in the removal percentage, followed by a sharp decrease for solutions with concentrations higher than 50 mg L^−1^. The initial concentration of CV and MG did not affect the removal efficiency of the frustules; in both cases, the removal was always higher than 90% (Figure 7a). For the isotherm, there was a strong positive correlation between adsorption capacity and equilibrium concentration of CR, CV, and MG in the solution (Figure 7b–d). Adsorption isotherms showed that the maximum adsorption capacity (q_max_) of the SZCZM1454 *H*. cf. *salinicola* frustules against the diazo dye is lower than against the basic dyes: 13.04 mg g^−1^ for CR and 41.97, 33.19 mg g^−1^ for CV and MG, respectively. 

Linear forms of Langmuir, Freundlich, Temkin, and Sips models (Figure S2) were applied to further understanding the possible adsorption mechanisms. The isotherm parameters as well as the coefficients of correlation and the normalized standard deviation are presented in Table 4. The equilibrium study showed that adsorption of different types of dyes follows different models: the Sips model is more applicable to CR adsorption data with closer to unity R^2^ and the lowest Chi-square values, while the most suitable model for the CV and MG adsorption onto the SZCZM1454 *H*. cf. *salinicola* frustules is the Freundlich model with R^2^ higher than 0.95 and χ^2^ less than 0.8.

## 3. Discussion

Table 5 shows the difference in growth parameters between different *Halamphora* spp. strains. The strain SZCZM1454 *H*. cf. *salinicola* has a lower specific growth rate and longer cultivation time, but the highest dry biomass yield and cell density than other strains reported (slightly lower than *H. coffeaeformis* from Argentina [6]). Furthermore, an increase in silicate concentration and salinity enhanced the biomass accumulation up to almost 1 g L^−1^ DW (Table 1), which is the highest biomass yield reported for any *Halamphora* spp. strain. The ability of diatoms to build the silicon frustules requires a sufficient concentration of silicate in the medium, and previous studies showed that silicon enrichment of the medium results in higher biomass yields [71,72]. 

Cultivation experiments with different temperature treatments revealed a significant difference in the biomass yield cultivated under 30 °C (Table 1). The SZCZM1454 *H.* cf. *salinicola* originates from Köyceğiz Lake in Turkey [10], which contains subaqueous hot springs [76]. Therefore, the strain could be considered thermophilic, hence the higher temperature requirements for cultivation. Similar results were obtained for a *Halamphora* sp. strain from a thermal spring in Tunisia, where the biomass accumulation increases with increasing temperature [9]. 

Scanning electron microscopy provided the surface topography of the frustules. Each frustule consists of two valves, and every valve has more than 200 areolae, thereby confirming the porous nature of the biosilica material. According to the IUPAC classification [77], pores with a diameter less than 2 nm are interpreted as micropores, pores with a diameter in the range of 2–50 nm are considered to be mesopores, and pores with diameters of 50 nm and larger are identified as macropores. The areolae could be considered as mesopores and macropores as they were 50–200 nm in diameter [78]. To study the microporous and mesoporous structure of the frustules, the BJH (Barrett–Joyner–Halenda) algorithm was applied. Pore diameter distribution (Figure S3) showed that the frustules have pores with a diameter from 3 nm to 35 nm, with an average pore diameter of 3.72 nm. Therefore, the frustule surface not only has areolae, but other micro and mesopores. Previous reports showed that the average pore diameter of biosilica was calculated as 3.93 nm for *P. trainorii* [79], 3–10 nm for *Thalassiosira punctigera,* 3.6–3.7 nm for *T. weissflogii* [80], and 4.61 nm for *Navicula australoshetlandica* [81]. These findings were confirmed by the low temperature nitrogen adsorption/desorption results: the isotherm for the SZCZM1454 *H*. cf. *salinicola* frustules could be classified as a combination of Type I and Type II isotherms with a Type H4 hysteresis loop, according to the IUPAC codification. Type I describes a microporous material with a small external surface, while Type II corresponds to macroporous or nonporous substances. The presence of the Type H4 hysteresis loop indicates the existence of slit-like pores in the sample [77]. 

Several authors have demonstrated that the specific surface area (BET) of different species of diatoms differs drastically, from 2 m^2^ g^−1^ and 4 m^2^ g^−1^ for *Skeletonema* sp. and *N. ramossisima,* respectively [82], and a 12–32 m^2^ g^−1^ range for *T. weissflogii* and *T. punctigera* [80], 30 m^2^ g^−1^ for *P. trainorii* [79] and 401.45 m^2^ g^−1^ for *N. australoshetlandica* [81]. Furthermore, [70] demonstrated that the specific surface area of the diatom frustules corresponds to the cleaning technique used and proposed the Sono–Fenton method, which increased the specific surface area from 14.71 m^2^ g^−1^ to 132.67 m^2^ g^−1^ for the *Cyclotella* sp. frustules. 

The presence of silica on the surface of diatoms was demonstrated by UV–vis spectroscopy. The same peak at 230 nm was recorded in both the sonicated biomass and the clean frustules. According to several reports [83,84,85], this peak reveals the presence of silica in both samples. The bands at 430, 495, and 676 nm shown in the sonicated biomass spectra indicate the presence of carotenoids and chlorophyll in the sample [86]. The band at 270 nm in the sonicated biomass sample could be explained by the presence of carbohydrates [87]. 

In order to determine the elemental composition of the frustules, an EDS analysis was performed. The spectra confirmed the UV–vis spectroscopy results and demonstrated distinct peaks of silicon (Si) and oxygen (O). The calculated atomic ratio O/Si of the frustules was 2.04; therefore, we can conclude that the SZCZM1454 *H*. cf. *salinicola* frustules were made of SiO_2_ [88]. The distinct peak of carbon (C) could indicate the presence of organic compounds within the frustules [89]. However, it is important to note that oxygen is too light to be properly indicated by EDS (there is a limitation for the elements with an atomic number below Na), and SEM oil pump contamination could also contribute to the observed carbon peak.

To analyse the frustule structure, the identification of functional groups on the surface is important. ATR-FTIR spectra showed a series of bands in the fingerprint region, which according to several reports [85,90,91,92], indicates bending (445 cm^−1^), symmetrical stretching (805 cm^−1^), and asymmetrical stretching (956, 1075, and 1150 cm^−1^) of the Si–O groups. According to [93] the vibration in the 565 cm^−1^ region could be due to the contribution of the siloxane rings. The adsorption peaks at 1380 and 1470 cm^−1^ are attributed to the bending vibrations of the N–H groups, indicating the presence of organic substances around the frustules [94,95]. Strong bands in the 2800–3000 cm^−1^ regions show stretching of the C–H groups [92]. Small IR vibrations are visible in the 1670, 1720, and 3660 cm^−1^ regions and they indicate the presence of the H–O–H deformation, the C = O stretching, and the O–H asymmetric stretching of the Si–OH group, according to several authors [85,90,92,94]. 

The X-ray powder diffraction analysis allowed us to study the crystalline structure of the sample. The results indicated the presence of amorphous silica in the sample, seen as a continuous elevation in the 2θ range from 10° to 35° [96]. Moreover, the results provided evidence for the presence of impurities in the biosilica sample: the pattern revealed the presence of calcium carbonate in the form of both vaterite and calcite [97]. 

Three stages could be distinguished in the TGA curve: the first one is related to the release of adsorbed water, the second one came from the decomposition of organic residue [98], and the third one indicates the decomposition of carbonaceous salts [98,99,100]. A more precise search revealed that the analysed sample probably had a significant amount of CaCO_3_, as the mass loss in the characteristic temperature range (590–740 °C) was 34.8% [97]. A further decrease in the mass at higher temperatures may come from the dehydration of the silanol groups.

Knowledge about the surface charge is important for two reasons. Firstly, the value of zeta potential indicates the stability of the suspension. The data from the literature indicates that stable and non-aggregating systems are characterized by absolute zeta potential values greater than +/−25 – +/−30 mV [101,102]. Secondly, zeta potential determines the adsorption properties of the material. For instance, a negatively charged surface will have a higher affinity to cationic compounds. It is noteworthy to mention that the zeta potential is dependent not only on their properties but can be manipulated through changes in the pH and ionic strength of the solution. Thus, both the stability and sorption properties of the material can be changed. The sample revealed a positive charge in the acidic conditions, which is not characteristic of pure silica [103]. The respective difference could be due to the presence of a high amount of impurities in the sample, as indicated by X-ray diffraction and thermogravimetric analysis. Moreover, at a pH of 7, the examined biosilica had a zeta potential of –25.1 mV, indicating the high stability of the system. Generally, the system is stable in a pH range of 7–12. These results highlight the dissimilarity in the sorption of the different types of dye: at a pH of 7, the frustule surface with the negative charge (contributed by the –SiO^−^, –COO^−^, and –NH_2_^−^ groups) interacts with the anionic CR (the –SO_3_^−^ and –NH_2_^−^ groups) with electrostatic repulsion, while the cationic CV and MG have the positive charge (=N^+^ groups) and interact with the frustules electrostatically, which resulted in a higher adsorption ability of the frustules for the cationic dyes.

The frustules of SZCZM1454 *H*. cf. *salinicola* showed a good adsorption ability. Removal of the diazo dye CR by the diatom frustules (74.9%) was comparable to activated carbon: 87.5% removal of 200 mg L^−1^ [48], as well as biosorbents; the microalgal waste showed 76.6% of removal of 50 mg L^−1^ after 120 min [57], pretreated *Scenedesmus obliquus* removed 41.15% of 20 mg L^−1^ CR [104], 87.66% removal of 20 mg L^−1^ for *Sargassum latifolium* waste [105], up to 88.7% of 100 mg L^−1^ CR removal by *Moringa* seed waste [66], 89.20% 55.04% of 100 mg L^−1^ CR for *Aspergillus fumigatus* and *Pseudomonas putida* mycelial pallets, respectively [106], and 15% and 98% removal of 50 mg L^−1^ for the unmodified and the modified diatomaceous earth, respectively [107]. At the same time, the maximum adsorption capacity of the frustules (q_max_ = 13.04 mg g^−1^) was lower than other sorbents: 493.8 mg g^−1^ for activated carbon [48], 316.46 mg g^−1^ for mycelial pellets [106], 202.9 mg g^−1^ for the *Chlorella vulgaris* biomass [108], 20.97 mg g^−1^ for *Sargassum latifolium* waste [105], 170.7 mg g^−1^ for *Moringa* seed waste [66], 55.5 mg g^−1^ for natural perlite [109], and 23.2 mg g^−1^ and 305.8 mg g^−1^ for the unmodified and the modified diatomaceous earth, respectively [107]. 

The efficiency of CV removal by the frustules (94.02%) corresponded to previous studies of algal biosorbents: 75% of 50 mg L^−1^ CV at a pH of 2 by modified *Spirulina* sp. [110], around 89% of 80 mg L^−1^ CV at a pH of 10 by *Laminaria japonica* [111], 93.04% of 20 mg L^−1^ CV at a pH of 7 by red seaweed [112], 98% of 5 mg L^−1^ CV at a pH of 3 by diatom *Skeletonema costatum* [67]. For natural siliceous materials, 99.9% by zeolite [113] and close to 100% by the diatomaceous earth [64]. The maximum adsorption capacity of the frustules (q_max_ = 41.97 mg g^−1^) was higher than the dye uptake of the *Skeletonema costatum* biomass (6.41 mg g^−1^) [67], the leaf biomass of *Calotropis procera* (4.14 mg g^−1^) [114], and fly ash zeolite (19.6 mg g^−1^) [115], which were almost the same as the adsorption capacity of the amino silica (40 mg g^−1^) [116] and kaolin (47.27 mg g^−1^) [61], and less than the capacity of modified *Spirulina* sp. (q_max_ = 101.87 mg g^−1^) [110], red seaweed (q_max_ = 150.14 mg g^−1^) [112], diatomaceous earth (q_max_ = 96.1 mg g^−1^) [64], natural zeolite (q_max_ = 177.75 mg g^−1^) and commercial activated carbon (q_max_ = 84.11 mg g^−1^) [113].

The diatom frustules of SZCZM1454 *H*. cf. *salinicola* demonstrated the highest removal efficiency (99.81%) against the basic dye MG compared to different adsorbents: commercially available powdered activated carbon showed 96% removal of 100 mg L^−1^ MG after 15 min [117], the xerogel activated diatomaceous earth presented up to 91.73% of removal of 10 mg L^−1^ [118], while diatomite from China showed up to 93.72% removal of 20 mg L^−1^ at a pH of 7 [65], fly ash removed only 64.42% of 20 mg L^−1^ MG from the solution [119], and the modified chitosan composite demonstrated 85% removal [120]. The maximum adsorption capacity of the diatom frustules against MG was calculated as 33.19 mg g^−1^, which is less than activated carbon (490.77 mg g^−1^ [121] and 222.2 mg g^−1^ [117]), the biomass of freshwater algae *Pithophora* sp. (117.65 mg g^−1^) [122], and brown marine algae *Turbinaria conoides* (66.60 mg g^−1^) [123]. It was, however, similar to zeolite (46.35 mg g^−1^) [124], tetrahedral silica (45.05 mg g^−1^) [125], and diatomite from China (23.64 mg g^−1^) [65], and higher than the xerogel activated diatomaceous earth (from 4.118 to 4.202 mg g^−1^) [118], fly ash (0.644 mg g^−1^) [119], and modified chitosan composite (4.8 mg g^−1^) [120]. The maximum adsorption capacities of SZCZM1454 *H*. cf. *salinicola* against CR, CV, and MG were smaller than other adsorbents (e.g., activated carbon) due to the relatively small specific surface area (S_BET_) of the frustules.

To understand the possible mechanism of adsorption of CR, CV, and MG onto the SZCZM1454 *H*. cf. *salinicola* frustules, several kinetic, diffusion, and isotherm models were applied. Parameters and correlation coefficients, calculated for each type of dye, showed that CV and MG adsorption are consistent with the Pseudo-Second Order model. The model highlighted the fact that the rate of CV and MG adsorption is controlled by chemisorption, involving covalent forces sharing or exchanging electrons between the adsorbent surface and the dye molecules. The CR adsorption process predominantly follows the Pore diffusion model – modified Intra particle diffusion model, which shows that the rate-controlling step in CR adsorption onto the SZCZM1454 *H*. cf. *salinicola* frustules is mostly through the diffusion of dye molecules onto pores inside the adsorbent particles [126]. 

Additionally, a widely used for determination of the main resistance to mass transfer the linearity test of Boyd’s model was applied. The plot for CR adsorption data passed through the origin, while the plots for CV and MG adsorption did not. Hameed et al. [127] stated that if the plot is nonlinear or linear, but does not pass through the origin, then film diffusion or chemical reaction control the adsorption rate, and if the plot is linear and passes through the origin, then the rate controlling step of mass transfer in this adsorption is through pore diffusion. We can conclude that CR adsorption was governed by pore diffusion, and for CV and MG adsorption onto the SZCZM1454 *H*. cf. *salinicola* frustules through the thin film (boundary layer), diffusion is the main resistance to mass transfer. 

The adsorption Isotherms were characterized by their shape: the CV isotherm was LS-shaped, and the CR and MG were mostly L-shaped [128]. The isotherm parameters showed that for CV and MG adsorption, the Freundlich model had the best fit, thus suggesting the multilayer formation of the basic dye onto the heterogenous sites on the surface of SZCZM1454 *H*. cf. *salinicola*, and CR adsorption followed the Sips model, which combines the Freundlich and Langmuir isotherms and describes the monolayer formation onto homogenous and heterogenous sites [129]. The adsorption in the Freundlich model is considered favorable, when 1 < *n* < 10, and the higher the *n*-value, the stronger the adsorption; therefore, CV (*n* = 1.18) and MG (*n* = 1.63) were favorably adsorbed onto SZCZM1454 *H*. cf. *salinicola* [130]. 

## 4. Materials and Methods

### 4.1. Chemicals

Malachite Green (99%, MW 364.911 Da), Crystal Violet (99%, MW 407.99 Da), and Congo Red (99%, MW 696.69 Da) were purchased from Hadron Scientific (Kielce, Poland). Thiamine hydrochloride (99%, MW 337.27 Da), biotin (>99%, MW 244.31 Da), vitamin B12 (>98%, MW 1355.37 Da), sodium hydroxide (>98%, MW 40.00), hydrochloric acid (37%, MW 36.46), and standard buffered solutions pH 2.0, 7.0, and 10.0 were supplied by Sigma-Aldrich (St. Louis, MO, USA). Hydrogen peroxide (30%, MW 34.01 Da), sodium nitrate (>99%, MW 84.99 Da), sodium dihydrogen phosphate monohydrate (>99%, MW 137.99 Da), sodium molybdate dihydrate (>99%, MW 241.95 Da), manganese (II) chloride tetrahydrate (>99%, MW 197.91 Da), and cobalt (II) chloride hexahydrate (>99%, DW 237.93 Da) were obtained from Chempur^®^ (Piekary Śląskie, Poland). Zinc sulfate heptahydrate (>99%, MW 287.54 Da), iron (III) chloride hexahydrate (>99%, MW 270.32 Da), EDTA disodium dihydrate (>99%, MW 372.24 Da), and copper (II) sulfate pentahydrate (>99%, MW 249.68 Da) were purchased from Scharlab (Barcelona, Spain). Nonahydrate sodium metasilicate (44–47.5% total solids, MW 284.19 Da) was supplied by Acros Organics, ThermoFisher Scientific (Waltham, MA, USA). The PhosVer^®^ 3 Phosphate Reagent Powder Pillow, Amino Acid F Reagent Powder Pillow, Citric Acid Powder Pillow, and Molybdate 3 Reagent Solution were provided by HACH-Lange (Loveland, CO, USA). Deionized water was obtained by using a Milli-Q^®^ purification system (Millipore Co., Bedford, MA, USA).

### 4.2. Batch Cultivation

The SZCZM1454 *Halamphora* cf. *salinicola* strain [10] was acquired from the Szczecin Diatoms Culture Collection (SZCZ), University of Szczecin, Institute of Marine and Environmental Sciences, Poland. For growth rate determination, the monoclonal culture was maintained in a 100 mL Erlenmeyer flask containing 35 ppt Guillard’s artificial seawater f/2 medium [131] (1 L of medium contained 880 µM NaNO_3_, 36 µM NaH_2_PO_4_ H_2_O, 106 µM Na_2_SiO_3_ 9H_2_O, trace metal: 0.08 µM ZnSO_4_ 7H_2_O, 0.9 µM MnSO_4_ H_2_O, 0.03 µM Na_2_MoO_4_ 2H_2_O, 0.05 µM CoCl_2_ 6H_2_O, 0.04 µM CuCl_2_ 2H_2_O, 11.7 µM FeCl_3_ 6H_2_O, 11.7 µM Na_2_EDTA 2H_2_O, vitamin B12, biotin and thiamine) under the following conditions: 20 °C constant temperature and 100 µmol s^−1^ m^−2^ white light under a under a 12:12 day/night light cycle in a plant growth chamber (FITO1400i, Biogenet, Poland) for 24 days. The nitrate concentration in the medium was measured before and after cultivation by the UV screening method (HACH-Lange), the orthophosphate by the Ascorbic Acid method (PhosVer 3^®^ powder pillows, HACH-Lange) and the silica content was monitored using the Heteropoly Blue method (Molybdate 3, Citric acid and Amino acid powder pillows, HACH-Lange). Cells were counted under an inverted microscope (Olympus CKX41, Olympus-Shinjuku, Tokyo, Japan) using a Malassez counting chamber (Marienfield, Germany) and the biomass was harvested by centrifugation at 3000 rpm followed by heat drying for 3 days at 50 °C. The regression curve was built, and the specific growth rate (µ) was calculated, using the following Equation (1) [132]:

B_t_ = B_0_ × e^μt^, (1)

where B_t_ is the biomass concentration at time (t) and B_0_ is the initial biomass concentration. Moreover, the influence of nutrient enrichment (5, 10, 15, and 20 times higher concentrations of NO^3−^, PO_4_^3−^, and SiO_3_^2−^), salinity (15, 20, and 45 ppt), temperature (15 and 30 °C), and illumination intensity (10, 50, and 150 μmol photons m^−2^ s^−1^) on the biomass accumulation in the late exponential phase was studied. For biosilica extraction, the strain SZCZM1454 was cultured in a 70 L cylindrical, air-lifted photobioreactor under optimized conditions.

### 4.3. Characterization of the Frustules Surface

To obtain the frustules, the biomass was harvested in the late exponential phase and exposed to a 30% H_2_O_2_ solution for 3 days with a thorough cleaning with ddH_2_O. The colorless frustules were heat dried at 50 °C for 3 days. A scanning electron microscope Hitachi SU8000 equipped with an EDS detector (Hitachi, Tokyo, Japan) was used for morphological as well as elemental composition studies of the cleaned frustules. The dried sample was sputter coated with a 10 nm thick gold layer. The measurements were performed with an accelerating energy of 30.0 kV. The sonicated in ddH_2_O control biomass and the cleaned frustules were subjected to the UV–Vis DR 6000 spectrophotometer (HACH-Lange) for optical measurements in the wavelength range of 200 to 900 nm in 10 mm cuvettes against a ddH_2_O (blank). For absorbances higher than 2, the solution was diluted with ddH_2_O and the dilution factors were considered in the presented graphs. The attenuated total reflection (ATR) mode on an Alpha FTIR spectrometer (Bruker Daltonics, Bremen, Germany) was used for the FTIR spectroscopic study of the cleaned frustules in the mid-infrared range (4000–400 cm^−1^). The low-temperature nitrogen adsorption/desorption isotherms were recorded on a Quantachrome Autosorb iQ at 77.35 K. Thermogravimetric analysis (TA/TGA) of the biosilica was performed using a TA Instrument type SDT 2960 (Artisan Technology, Champaign, IL, USA) at a 0–1100 °C temperature range, 100 mL min^−1^ air flow rate, and 10 °C min^−1^ heating rate. The analysis data for TA/DTA was proceeded with the use of TA Universal Analysis software (version 4.5A, TA Instruments, New Castle, DE, USA). Zeta potential change over the pH was analysed with a Malvern Zetasizer NanoZS (Malvern Instruments, Malvern, UK) using dedicated cuvettes DTS1070 (Malvern Instruments, Malvern, UK). The analysis was made considering the Smoluchowski approximation, with the automatic selection mode of voltage and number of runs, and in the pH range of 2.0–12.0. The diatom frustules were suspended in deionized water at a concentration of 0.5 mg mL^−1^. The solutions of 0.1M NaOH and 0.1M HCl were used for pH adjustment of the suspension. The pH of the suspension was measured using a FiveEasy Plus pH-meter (Mettler Toledo) with a combined electrode with glass membrane and Ag/AgCl reference system (Mettler Toledo). The pH-meter was calibrated using standard buffered solutions with a pH of 4.0, 7.0, and 10.0 before carrying out measurements. The X-ray diffraction analysis was made with an X’Pert Pro Analytical diffractometer (Phillips, Erlangen, Germany) with CuKα radiation source and Ni filter. The X-ray diffraction pattern analysis was obtained from the XRD Malvern Panalytical software (version 1.5a, Almelo, The Netherlands) in the 2θ range from 5 to 120° with a scan step size of 0.0167°.

### 4.4. Batch Adsorption Experiments

Three types of dye–Congo Red (CR), Crystal Violet (CV), and Malachite Green (MG)–were used in batch adsorption experiments. The dried frustules (20 ± 0.5 mg) were exposed to 15 mL of 20.0 mg L^−1^ CR, CV, and MG solutions (pH–7) in a Falcon tube and stirred at 3000 rpm at 20 °C for 4 h. The kinetics of adsorption were recorded spectrophotometrically (UV-Vis DR 6000, HACH-Lange) at 499 nm (CR), 586 nm (CV), and 616 nm (MG) at different time points: 5, 10, 15, 30, 45, 60, 90, 120, 180, and 240 min. The influence of different initial concentrations of dye was investigated by measuring 10 mg of the cleaned frustules in Falcon tubes containing various concentrations (5, 10, 15, 20, 50, and 100 mg L^−1^) of CR, CV, and MG. After the mixture reached equilibrium (120 min for CR and MG and 60 min for CV), the removal was recorded spectrophotometrically, as mentioned above. The quantity of adsorbed MB by the frustules was calculated as follows (Equation (2)):

q_t_ = V × (C_0_ − C_t_)/m, (2)

where q_t_ is the MB adsorbed on the frustules (mg g^−1^) at a given time (t); C_0_ and C_t_ are the concentrations of the MB at the start and at the given time point (mg L^−1^), respectively; V is the solution volume (L); and m is the biosilica dosage (g). 

The percentage of MB removal (%) was calculated as in Equation (3):

Removal (%) = 100% × (C_0_ − C_t_)/C_0_, (3)

where C_0_ and C_t_ are the concentrations of the MB at the start and at the given time (t), respectively (mg L^−1^). 

To understand the possible mechanisms of adsorption, the linearized forms of Pseudo-First Order, Pseudo-Second Order, Elovich kinetic models, and Langmuir, Freundlich, Temkin, and Sips isotherm models were applied (Table S1). 

### 4.5. Data Analysis

Batch cultivation experiments were conducted in duplicate. The zeta potential measurements were performed in triplicates. Batch adsorption experiments were conducted without replication. The figures show the mean values and standard deviation. The significance of differences between groups was analysed using a one-way ANOVA analysis with Tukey HSD test. A significant difference between two groups was declared if *p* < 0.05. The batch growth experiment figures, UV–Vis, Tauc plot, FTIR spectra, batch adsorption spectra, and models were plotted using MS Excel software (version 16.73). The EDS spectra was obtained using NSS ThermoScientific software (version 1.0). The nitrogen adsorption/desorption isotherms were acquired using Quantachrome Instruments software (version 1.11). The applicability of the kinetic, diffusion, and isotherm models was validated using the Chi-square (χ^2^), which is defined as (Equation (13)):

χ^2^ = S[(q_cal_ − q_exp_)^2^/q_exp_], (4)

where q_exp_ and q_cal_ (mg g^−1^) are the experimental and calculated adsorption capacity value, respectively.

## 5. Conclusions

The present study explored the ability of the mesoporous nanostructured siliceous frustules extracted from the diatom culture of *Halamphora* cf. *salinicola* Levkov and Díaz to perform as a novel eco-friendly adsorbent for different classes of dyes: an anionic diazo Congo Red (CR), a cationic basic Crystal Violet (CV), and Malachite Green (MG). This diatom strain is known to produce high amounts of neutral lipids, unsaturated fatty acids, fucoxanthin, and chrysolaminarin, and could be used in biodiesel, aquaculture, cosmetics, and pharmaceutics. The diatom wastes from these industries could be purified and used in wastewater treatment. This study for the first time introduced the diatom frustules as a novel efficient adsorbent of the basic and diazo dyes from wastewaters. This method resulting in no more than 75% removal of the diazo dye (CR) that decreased with increased concentrations, with the maximum adsorption capacity of 13.04 mg g^−1^, while removal of the basic dyes (CV and MG) by diatomaceous earth was higher than 90% in every experiment, with higher adsorption capacity (41.97 and 33.19 mg g^−1^, respectively) than several other known biosorbents.

## Figures and Tables

**Table 1:** The dry biomass yield (g L^−1^ DW) of SZCZM1454 *H*. cf. *salinicola* under different cultivation parameters.

**Nitrate, mM**					**Phosphate, mM**				
**0.88**	4.41	8.82	13.23	17.64	0.04	0.18	0.36	0.54	0.72
**0.610 ±** **0.0206**	0.556 ± 0.0295	0.627 ± 0.0300	0.670 ± 0.0899	0.641 ± 0.0457	0.646 ± 0.0321	0.631 ± 0.0226	0.604 ± 0.0707	0.701 ± 0.0074	0.704 ± 0.0530
**Silicate, mM**					**Temperature, °C**				
**0.11**	0.53	1.06	1.59	2.12	15	20	30		
**0.501 ±** **0.0045**	0.644 ± 0.0274	0.646 ± 0.0442	0.825 ± 0.0093	0.964 ± 0.0471	0.431 ± 0.0015	0.480 ± 0.00402	0.700 ± 0.0346		
**Light, ** **µ** **mol s^−1^ m^−2^**					**Salinity, ppt**				
**10**	50	100	150		15	20	35	45	
**0.342 ±** **0.0619**	0.712 ± 0.0527	0.547 ± 0.0511	0.641 ± 0.0436		0.388 ± 0.0779	0.355 ± 0.1576	0.603 ± 0.0751	0.969 ± 0.0228	

**Table 2:** Porous structure of the surface of the SZCZM1454 *H*. cf. *salinicola* frustules.

Specific Surface Area (m^2^ g^−1^)	Pore Volume (cm^3^ g^−1^)	Pore Diameter Distribution (nm)
**26.922**	0.175	3–35

**Table 3:** Parameters of kinetic and diffusion models for CR, CV, and MG adsorption onto the SZCZM1454 *H*. cf. *salinicola* frustules.

**Type of Dye**	**Pseudo First Order**				**Pseudo Second Order**				**Boyd’s**	
	q_1_, mg g^−1^	k_1_, min^−1^	R^2^	χ^2^	q_2_, mg g^−1^	k_2_, g (mg min)^−1^	R^2^	χ^2^	R^2^	χ^2^
**CR**	11.95	0.008	0.947	32.41	14.64	0.0005	0.859	1.47	0.947	16.91
**CV**	1.64	0.003	0.142	75.5	9.36	0.4491	0.954	3.15	0.957	0.16
**MG**	3.87	0.010	0.720	57.59	11.64	0.0145	0.999	0.25	0.996	0.51
**Type of dye**	**Intra particle diffusion**				**Pore diffusion**				**q_exp_, mg g^−1^**	
	K_wm_, mg (g min^0.5^)^−1^	B, mg g^−1^	R^2^	χ^2^	Δβ	k_β_	R^2^	χ^2^		
**CR**	0.707	1.203	0.935	1.82	0.677	0.0003	0.951	1.37	8.81	
**CV**	0.146	7.813	0.267	1.43	0.088	0.0088	0.479	1.00	10.44	
**MG**	0.441	5.906	0.759	1.25	0.201	0.005	0.907	0.61	11.86	

**Table 4:** Parameters of four linear isotherm models for CR, CV, and MG adsorption onto the SZCZM1454 *H*. cf. *salinicola* frustules.

Type of Dye	Langmuir				Freundlich				Temkin				Sips					q_exp_, mg g^−1^
	q_max_, mg g^−1^	K_L_, L mg^−1^	R^2^	χ^2^	K_F_, (mg g^−1^) (mg L^−1^)^-n^	n	R^2^	χ^2^	b, J mol^−1^	K_m,_ L g^−1^	R^2^	χ^2^	q_m_, mg g^−1^	K_S_ [(mg L^−1^)^−1/n^]	n	R^2^	χ^2^	
CR	87.72	26.22	0.018	5.05	3.66	0.94	0.786	10.02	549.6	0.33	0.916	2.69	13.4	0.08	0.47	0.950	1.26	13.04
CV	114.9	0.06	0.473	16.82	7.04	1.25	0.977	0.80	189.9	1.86	0.923	3.74	42.1	0.53	0.39	0.662	18.92	41.97
MG	41.5	753.1	0.938	1.31	10.39	1.63	0.999	0.06	384.4	11.0	0.874	19.02	35.0	0.77	0.91	0.900	3.75	33.19

**Table 5:** Growth parameters of different *Halamphora* strains cultivated under standard conditions.

Strain	Origin of Strain	Cell Density per mL	Biomass DW (g L^−1^)	Specific Growth Rate	Days of Cultivation	Ref.
*H. coffeaeformis*	Bahía Blanca Estuary, Argentina	1.40 × 10^5^		1.47 div d^−1^	7 days	[4]
*H. coffeaeformis*			0.3		22 days	[7]
*H. coffeaeformis*		10.0 × 10^5^	0.43	2.03 div d^−1^	13 days	[12]
*H. coffeaeformis*		7.8 × 10^5^	0.18	0.92 div d^−1^	6 days	[8]
*H. coffeaeformis*		37.0 × 10^5^ in PBR	0.64	0.4 div d^−1^	11 days	[6]
*H. luciae*		417 cells in mm^−2^		0.29 div d^−1^	18 days	[73]
UTCC58 *H. coffeaeformis*		2.51 × 10^5^		Max: 2.01 div d^−1 ^ Mean: 1.04 div d^−1^	8 days	[74]
*H. coffeaeformis*	Kelley’s Slough, ND, USA			1.01 div d^−1^	The growth rate was calculated after 48 h	[5]
*H. subturgida*	Sawhill Pond, CO, USA			0.26 div d^−1^		
*H. pertusa*	Blue Lake, UT, USA			1.33 div d^−1^		
*H. turgida*	Blue Lake, UT, USA			1.16 div d^−1^		
*H. oligotraphenta*	Sawhil Pond, CO, USA			0.33 div d^−1^		
*H*. cf. *borealis*	Guana River, FL, USA			0.98 div d^−1^		
SB1 MK575516.1 *Halamphora* sp.	Sfax Solar Saltern pond, Tunisia	8 × 10^5^			10 days	[75]
SZCZM1454 *H*. cf. *salinicola*	Köyceğiz Lake, Turkey	24.6 ± 0.04 × 10^5^	0.73 ± 0.047	0.081 d^−1^ 0.113 div d^−1^	27 days	This study

## Data Availability

The authors confirm that the data supporting the findings of this study are available within the article and its Supplementary Material. Raw data that supports the fundings of this study are available from the corresponding author, upon reasonable request.

## Supplementary Materials

The following supporting information can be downloaded at: https://www.mdpi.com/article/10.3390/md21050312/s1, Figure S1: Adsorption kinetic and diffusion studies: (a) Pseudo-First Order; (b) Pseudo-Second Order; (c) Intra-particle diffusion; (d) Pore diffusion model (experimental data: orange circles–CR, violet squares–CV, green triangles–MG; calculated data: Pseudo-First Order–dashed line, Pseudo-Second Order–dash dotted line, Intra-particle diffusion–dotted line, Pore diffusion–double-dash dotted line); Figure S2: Adsorption equilibrium study: Langmuir model for (a) CR, (b) CV, (c) MG; Freundlich model for (d) CR, (e) CV, (f) MG; Temkin model for (g) CR, (h) CV, (i) MG; and Sips model for (j) CR, (k) CV, (m) MG (experimental data: orange circles–CR, violet squares–CV, green triangles–MG; calculated data: Langmuir–dashed line, Freundlich–dash dotted line, Temkin–double dash dotted line, Sips–dotted line); Figure S3. Pore size distribution of the diatom frustules of SZCZM1454 *H*. cf. *salinicola*; Table S1. Linearized forms of kinetic and isotherm models’ equations and parameters [133,134,135,136,137,138,139,140,141].
