# Supplementary material for: Removal of the Basic and Diazo Dyes from Aqueous Solution by the Frustules of Halamphora cf. salinicola (Bacillariophyta)
Source: Mar Drugs. 2023 May 19;21(5):312. doi: 10.3390/md21050312 (PMC10223004; doi:10.3390/md21050312)
Supplement: Supplementary file 1 [file marinedrugs-21-00312-s001.zip › marinedrugs-2413889-supplementary.pdf]

SUPPLEMENTARY MATERIALS

Article

Removal of the Basic and Diazo Dyes from Aqueous Solution by the Frustules of *Halamphora* cf. *salinicola* (Bacillariophyta)

Aleksandra Golubeva[1*], Piya Roychoudhury[1], Przemysław Dąbek[1], Oleksandra Pryshchepa[2], Paweł Pomastowski[2], Jagoda Pałczyńska[3], Piotr Piszczek[3], Michał Gloc[4], Renata Dobrucka[4,5], Agnieszka Feliczak-Guzik[6], Izabela Nowak[6], Bogusław Buszewski[7,8] and Andrzej Witkowski[1*]

[1] Institute of Marine and Environmental Sciences, University of Szczecin, Mickiewicza 16a, 70-383 Szczecin, Poland; alexandra.golubeva@phd.usz.edu.pl (A.G); piyaroychoudhury2@gmail.com (P.R.); przemyslaw.dabek@usz.edu.pl (P.D.); andrzej.witkowski@usz.edu.pl (A.W.).
[2] Centre for Modern Interdisciplinary Technologies, Nicolaus Copernicus University, Wileńska 4, 87-100, Toruń, Poland; pryshchepa.alexie@gmail.com (O.P.); pomastowski.pawel@gmail.com (P.P.).
[3] Department of Inorganic and Coordination Chemistry, Faculty of Chemistry, Nicolaus Copernicus University in Toruń, Gagarina 7, 87-100 Toruń, Poland; 296600@stud.umk.pl (J.P.); piszczek@umk.pl (P.P).
[4] Faculty of Materials Science and Engineering, Warsaw University of Technology, Wołoska 141, 02-507 Warsaw, Poland; michalgloc@wp.pl (M.G.); renata.dobrucka@pw.edu.pl (R.D.).
[5] Department of Industrial Products and Packaging Quality, Institute of Quality Science, Poznań University of Economics and Business, al. Niepodległości 10, 61-875 Poznan, Poland; renata.dobrucka@ue.poznan.pl (R.D.).
[6] Department of Applied Chemistry, Faculty of Chemistry, Adam Mickiewicz University, Uniwersytetu Poznańskiego 8, 61-614, Poznań, Poland; agnieszka.feliczak-guzik@amu.edu.pl (A.F-G.); nowakiza@amu.edu.pl (I.N.).
[7] Department of Environmental Chemistry and Bioanalysis, Faculty of Chemistry, Nicolaus Copernicus University, Gagarina 7, 87-100 Toruń, Poland; bbusz@umk.pl (B.B.).
[8] Prof. Jan Czochralski Kuyavian-Pomeranian Research & Development Centre, Krasińskiego 4, 87-100 Toruń, Poland; bbusz@umk.pl (B.B.).
* Correspondence: alexandra.golubeva@phd.usz.edu.pl, Tel.: +48-534-030-336; andrzej.witkowski@usz.edu.pl

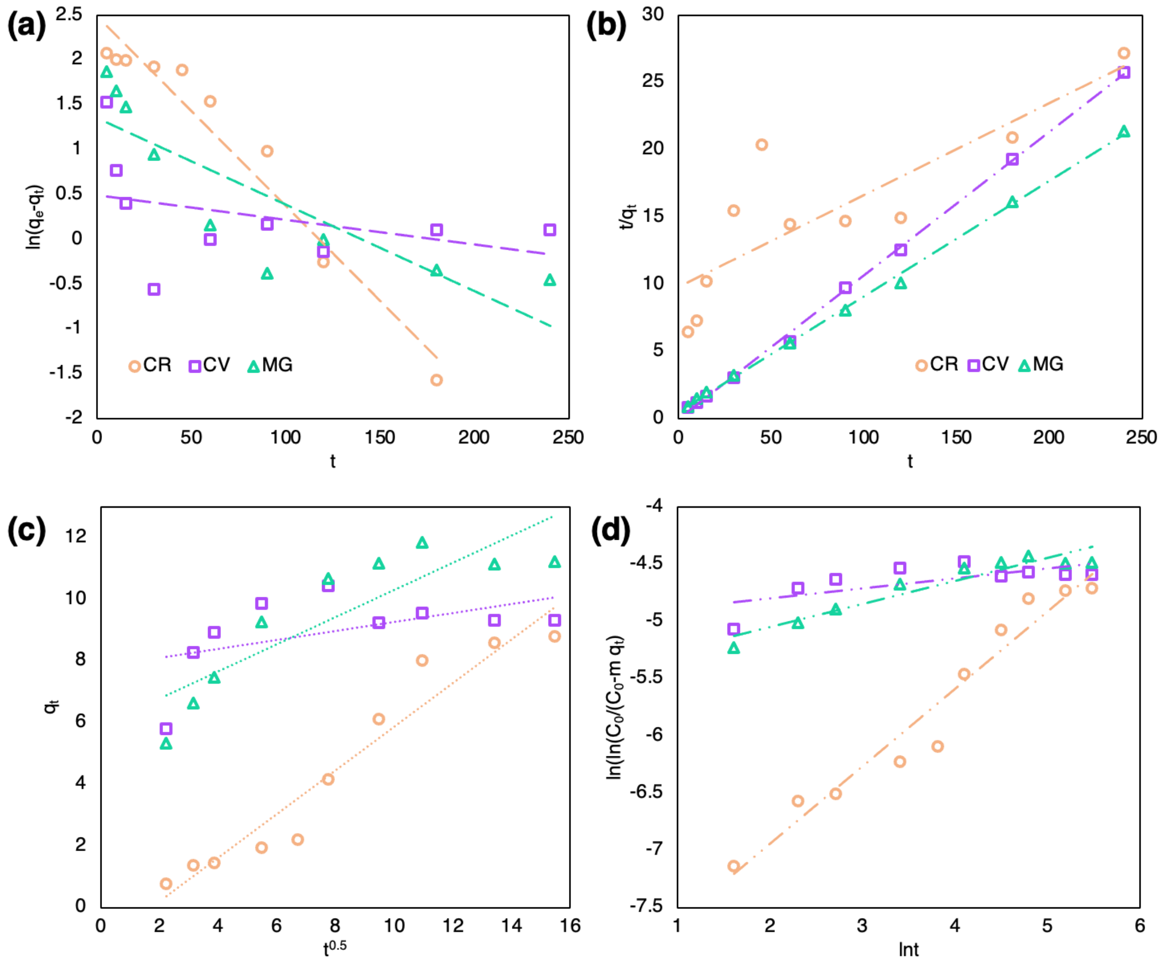

Figure S1. Adsorption kinetic and diffusion studies: **(a)** Pseudo-First Order; **(b)** Pseudo-Second Order; **(c)** Intra-particle diffusion; **(d)** Pore diffusion model (experimental data: orange circles – CR, violet squares – CV, green triangles – MG; calculated data: Pseudo-First Order – dashed line, Pseudo-Second Order – dash dotted line, Intra-particle diffusion – dotted line, Pore diffusion – double-dash dotted line).

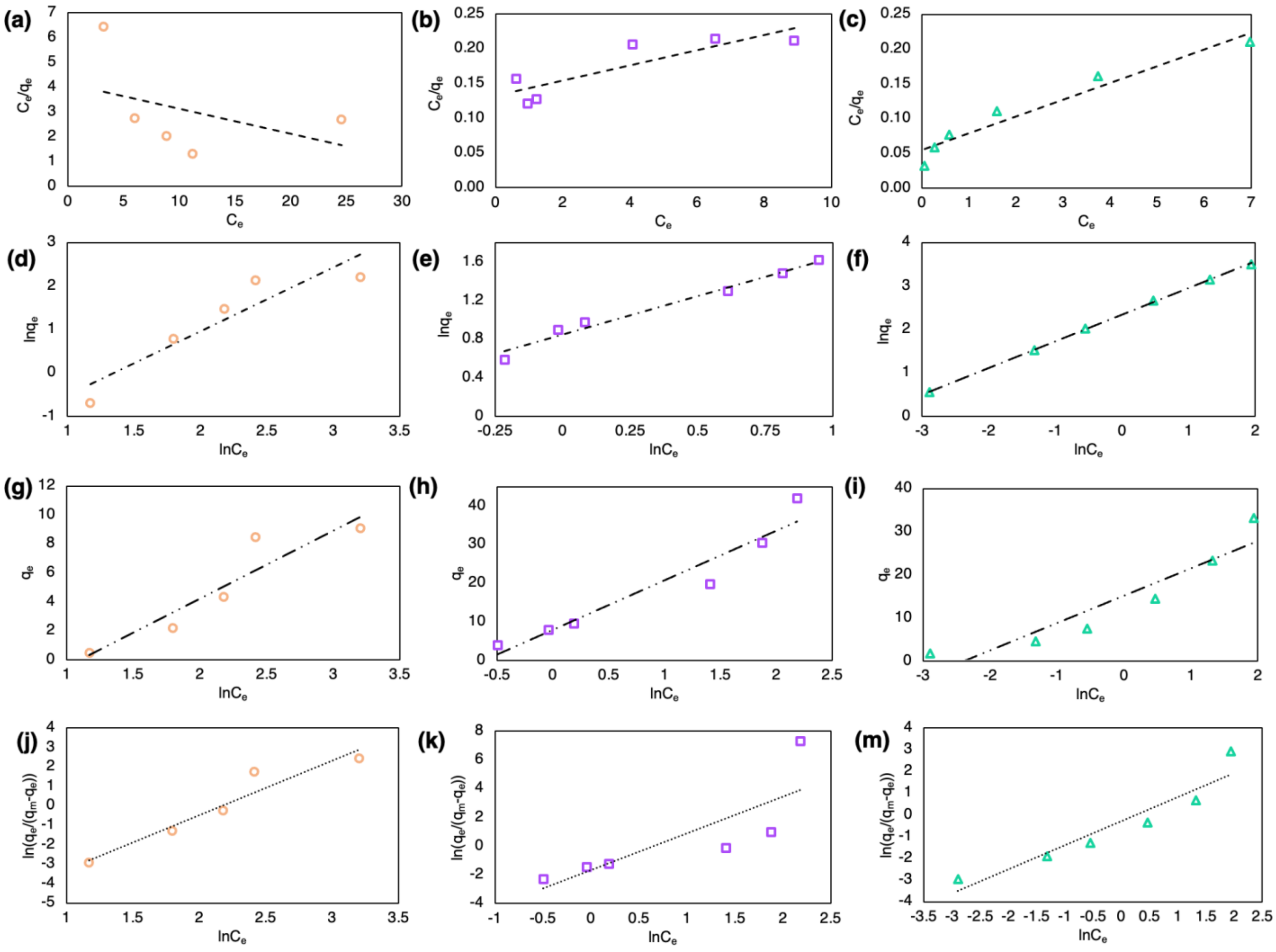

Figure S2. Adsorption equilibrium study: Langmuir model for **(a)** CR, **(b)** CV, **(c)** MG; Freundlich model for **(d)** CR, **(e)** CV, **(f)** MG; Temkin model for **(g)** CR, **(h)** CV, **(i)** MG; and Sips model for **(j)** CR, **(k)** CV, **(m)** MG (experimental data: orange circles – CR, violet squares – CV, green triangles – MG; calculated data: Langmuir – dashed line, Freundlich – dash dotted line, Temkin – double dash dotted line, Sips – dotted line)

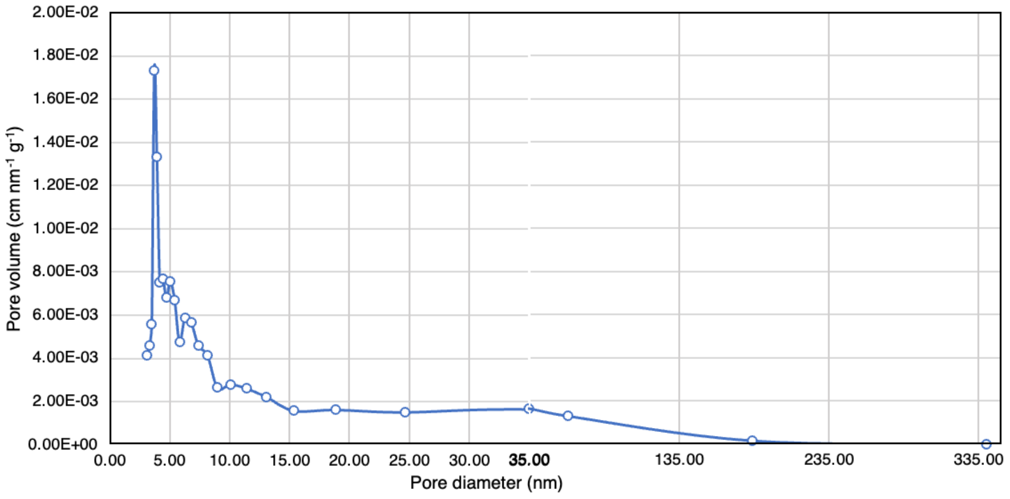

Figure S3. Pore size distribution of the diatom frustules of SZCZM1454 *H.* cf. *salinicola*

Table S1. Linearized forms of the kinetic and isotherm models' equations and parameters

Models	Equation		Description of parameters	Ref.
**Kinetic study**				
Pseudo-first order	$\ln(q_1 - q_t) = \ln q_1 - k_1 t$	(1)	q_t (mg g^{-1}) – amount adsorbed at given time (t, min)<br>q_1 (mg g^{-1}) – adsorbent capacity at equilibrium<br>k_1 (min^{-1}) – Pseudo-first order constant rate	[133]
Pseudo-second order	$\frac{t}{q_t} = \frac{1}{k_2 q_2^2} + \frac{1}{q_2}$	(2)	q_2 (mg g^{-1}) – adsorbent capacity at equilibrium<br>k_2 (g (mg min)$^{-1}$) – Pseudo-second order constant rate<br>α (mg (g min)$^{-1}$) – initial adsorption rate<br>β (mg g^{-1}) – desorption constant	[134]
**Diffusion study**				
Boyd's	$B_t = -0.4977 - \ln(1 - \frac{q_t}{q_e})$	(3)	q_e (mg g^{-1}) – amount adsorbed at equilibrium<br>B_t – mathematical function of F<br>F – the fraction of metal ion adsorbed at any time (t)	[135]
Intra particle diffusion	$q_t = k_{wm} t^{0.5} + B$	(4)	q_t (mg g^{-1}) – amount adsorbed at given time (t, min)<br>K_{wm} (mg (g $min^{0.5}$)$^{-1}$)– Intra-particle diffusion rate constant	[136]
Pore diffusion	$\log\log\left(\frac{C_0}{C_0 - mq_t}\right) = \log\left(\frac{mK_\beta}{2.303V}\right) + \Delta\beta \log t$	(5)	B (mg g^{-1}) – intercept<br>C_0 (mg L^{-1}) – initial concentration of MB<br>m (g) – mass of adsorbent g<br>Δβ and K_β - Bangham constants<br>V (mL)– volume of solution	[137]
**Equilibrium study (isotherms)**				
Langmuir	$\frac{C_e}{q_e} = \frac{1}{K_L Q_{max}} + \frac{C_e}{Q_{max}}$	(6)	q_e (mg g^{-1}) – adsorption capacity at equilibrium<br>C_e (mg L^{-1}) – concentration of MB dye at equilibrium	[138]
Freundlich	$\ln q_e = \ln K_F + \frac{1}{n} \ln C_e$	(7)	q_{max} (mg g^{-1}) – maximum monolayer adsorption capacity of Langmuir	[139]
Temkin	$q_e = \frac{RT}{b} \ln C_e + \frac{RT}{b} \ln K_m$	(8)	K_L (L mg^{-1}) – Langmuir constant	[140]
Sips	$\ln\left(\frac{q_e}{q_m - q_e}\right) = \frac{1}{n} \ln C_e + \ln K_S^{\frac{1}{n}}$	(9)	K_F [(mg g^{-1}) (mg L^{-1})$^{-n}$] – Freundlich constant<br>n – dimensionless Freundlich intensity parameter<br>R (J [mol K]$^{-1}$) – universal gas constant<br>T (K) – temperature<br>b (J mol^{-1}) – Temkin constant related to sorption heat<br>Km (L g^{-1}) – Temkin isotherm constant<br>q_m (mg g^{-1}) – the Sips maximum adsorption capacity<br>K_S [(mg L^{-1})$^{-1/n}$] – the Sips equilibrium constant<br>n – the exponent of Sips where $0<1/n\leq1$	[141]
